# Pathological Prescribing: “A Science Falsely So-called”

**Published:** 1881-07

**Authors:** E. J. Lee

**Affiliations:** Philadelphia


### PATHOLOGICAL PRESCRIBING: A "SCIENCE FALSELY SO-CALLED."

E. J. Lee, M. D., Philadelphia.

There have been from Hahnemann's day to the present, those in the homœopathic school, who desired to base their prescriptions upon the pathological condition, presumed to be present in a case under treatment. These would-be pathologists, embrace two classes—the one holding to this view from a lazy desire to make homœopathic prescribing an easy routine affair; the other, from a belief that pathology is the only true basis upon which they could build a rational therapeutics. In other words, one class is lazy and insincere; the other, diligent and sincere; yet both are, as we think, wrong. To the first class, we have naught to say, unless to repeat Hahnemann's warning, that "in a science in which the welfare of mankind is concerned, any neglect to make ourselves masters of it, is a *crime;*" to the second, we say: while respecting the opinions of all sincere and diligent laborers in the medical vineyard, we nevertheless believe the facts of science are against your views.

But a few words as to pathology in the Old School. It has been made much of by the allopath, and why? because, forsooth, he has no system of therapeutics worthy to be called scientific.

To cure disease, says he, we must know its nature; we must learn what each disease is, its causes, its characteristics and its conditions; then, and only then, can we rationally combat it. "Pathology dictates the maxims of rational practice," says Aitkin. This is, in brief terms, about the allopathic idea of the practical scope of pathology; and very plausibly it reads, but can one act on it? Is it true even when judged by allopathic practice? Do we know anything of the internal nature of disease?

If pathology is the only rational basis for scientific therapeutics, and if it be, at present, anything approaching an exact science, then surely those diseases whose pathology is considered best-known, should be most amenable to treatment; and conversely, those whose pathology is unknown, or especially dubious, should be but poorly handled. We all know that this relation between pathological knowledge and curative ability does not exist in the Old School. Let any doubting Thomas briefly review in his own mind a few diseases whose pathology is considered best known, and see if the curative power of that school has increased with its boasted pathological knowledge. To be fair, let him take almost any of the acute diseases, these being considered definite in their course and self-limited as to time; to judge allopathy upon its treatment of acute disease, is to give them an opportunity to show up their best work; to judge them upon their chronic cases, would be really *cruel.* To any one who thus briefly reviews allopathic practice of to-day, judging them out of their own records, it must be evident that pathology has not advanced them to a better curative skill.

If then, pathology does not afford allopathy the great assistance in curing that is claimed for it, it is well for the homœopath to ask, how does it aid us, and what is the proper sphere under our law? No one will deny that the thoroughly equipped homœopathic physician should be well educated in physiology, diagnosis and pathology, as well as in therapeutics, though the latter should be the chief corner-stone of medical education; all these to be used under and subject to our law. He who places any branch of medicine in an improper sphere, or to a wrong use, *misuses and perverts* it. He who misuses pathology cannot justly decry him who neglects its use altogether. Of the two errors, in our school, the abuse of pathology is the greater. Every branch of medicine has its proper sphere and use under our law; in that place it does much good; out of it, incalculable harm.

Said the late Prof. J. H. P. Frost*: "In its full and proper sense, pathology includes *all* that can be discovered of the patient's deviation from the normal standard of health; and comprehends alike *all the 'symptoms,' morbid conditions, their consequences and their causes.* Such pathology (which alone is worthy of the name of science,) becomes the perpetual study, *in the living subject,* of the homœopathician, and this all the more as he renders himself liable to be called a 'symptom-coverer.' This pathological condition does not exclude *post-mortem* examinations; it may end with them, but it never begins with them. It embraces alike the purely *subjective* or *sensational* symptoms; all *physiological* or *functional* deviations, and all *objective* or *external* morbid changes in form or color, in structure and in tissue. And if the practitioner of the 'symptom-method' overlook any of these causes, indications, or consequences of disease; if he fail to 'render unto Cæsar the things that are Cæsar's,' and neglect to give to *each class* and *particular evidence* of patholological deviation *its* JUST *value,* in making his prescription, his diagnosis and his prognosis, he will come to grief, and his patient with him. Indeed, we think it cannot but be obvious to every intelligent and candid mind, that no class of physicians more anxiously study and weigh the *just value and due relative importance* of pathological conditions, and consequently that none are more thorough students of pathology, properly so-called, than are those of the 'symptom-method' persuasion."

That learned and veteran homœopathist, P. P. Wells, M. D.† said: "But if the symptoms are the only guides to the selection of the curative, what becomes of the vaunted pathology of which we hear so much, and so often, from those who are slightly informed as to its nature, place or importance in our practical duties. To guard against the *wrong use* of this valuable science was another occasion for giving us this eighteenth section [of *The Organon*]. To put it

---

* See *Hahnemannian Monthly*, vol, 4, pp. 127-128.

† See January number of this Journal, p. 18.

[pathology] as a teacher in the selection of curatives, to the exclusion of the symptoms from that function, is to put it where it has no place in a rational system of healing, certainly none under the control of a natural law, which discloses the curative relationship as existing in the similarity between the symptoms of the drug and the disease. Where, then, is the practical use of this so highly prized science of pathology? In the duty of prescribing for the sick, its use is limited to aiding a right understanding of the nature and value of the symptoms revealed in the case in hand. Beyond this it has no function in the process of prescribing. Pathology, to illustrate, teaches a difference between inflammations and neuralgias. Both are attended by pains of the severest kind, but this science teaches that these have a different significance and often different importance, as the case in hand belongs to one class or the other. A knowledge of the science of pathology will enable us to relegate our case to its proper class, and there its function ceases. It cannot go beyond this; and having decided the case a neuralgia, say the remedy is *Aconite* or *Bell.* or *Bry.* or *Colocyn.* or *Hyosc.* or *Lach.* or *Merc.* or *Nux* or *Puls.* or *Rhus* or *Spig.*, or either of the many remedies which a given case may demand for its cure under the law. To attempt to give to this science this decision [*i. e.*, the choice of the remedy,] is to impose on it a function wholly out of the sphere of its legitimate use."

The lamented Carroll Dunham wrote:*

"Physiology and pathology themselves teach us that the science of pathology can in no sense serve as a basis or foundation for the science of therapeutics." Again: "But these advances in pathology, great as they have been, have not altered the relation which the phenomena of natural disease bear to those of drug disease. These phenomena respectively, whether rudely apprehended, or clearly and fully understood in all their relations and inter-dependencies, still bear the same relation to each other expressed by the law *Similia Similibus Curantur.* And we can imagine no possible development of the sciences of pathology and pathogenesy which could alter this relation."

Thus we see that the pathological condition of any case is included in the totality of the symptoms of that case. As a *part* of this totality, the pathological state is known and given its full value; as a something outside of, and separate from this totality it has no place nor function, save to do harm.

The real homœopath is a student of *minute* pathology; the allopath

* See "Homœopathy, the Science of Therapeutics," p. 28.

and his homœopathic imitator are students of superficial pathology. To illustrate, to the true homœopath every case of the same (nosological) disease is a study, to the allopath it is not so. To the real homœopath every syphilitic ulcer is a study; he notes its size, color, rapidity and shape of growth, its discharges and its general concomitant symptoms; to the allopath, and his homœopathic imitator, a chancre is a chancre, and for it he has his specific. To the real homœopath every case of pneumonia is a separate study, though he knows they are all of inflammatory nature, and in their gross features very similar; but he is not satisfied with a superficial knowledge of these gross features; he dives deeper, and then he discovers that each case of pneumonia is different from every other. These minute differences are to the true homœopath the guides to the selection of the proper remedy.

Hypothesis has no part nor lot in the homœopathic prescription; the homœopath does not attempt to translate the simple, truthful language of the symptoms into the ever changing, and always unintelligible, jargon of pathological diagnosis. A diagnosis of the symptoms of any given case might indeed point to fatty degeneration of the heart, or to a cirrhosis of the liver, or to some other artificial classification; nevertheless, the true homœopath administers the remedy indicated by the totality of the symptoms, not stopping to ascertain whether or no that remedy has ever caused fatty degeneration or cirrhosis. Any attempt at a pathological basis, for homœopathic prescriptions, must at once exclude mental and subjective symptoms, and these are often our surest guide to a proper selection, even though they be pathologically insignificant.

Having endeavored to briefly outline the sphere and use of pathology as a part of that totality of symptoms, which our law alone recognizes as the true basis for a correct homœopathic prescription, let us examine a few of the arguments adduced by those homœopaths who believe that pathology is the only true basis for therapeutics.

One of these would-be pathologists, whom we select as a specimen of his class (not, indeed, because of any especial merit on his part), writes: "*Muriatic acid*, the acknowledged remedy for zymotic blood-poisoning, when debility, with erethism, prevails, gives us bitter putrid eructations, gulping up of contents of stomach into œsophagus, sometimes going down again; empty sensation in stomach, extending through whole abdomen, but no hunger; morbid longing for alcoholic drinks; vertigo, with nausea; heaviness in occiput, with obscure sight, worse with effort to see—all symptoms hinting squarely to nervous debility."

"*Nitric acid: Liver and spleen enormously enlarged* and deranged is the key-note to the dyspepsia, curable by Nitric Acid." (Then follow some general dyspeptic symptoms).

"*Lactic acid* is frequently prescribed in the Old School in atonic dyspepsia, as well as in irritative dyspepsia, and cases of acidity and heartburn are quickly relieved if given before meals." (Here, too, follow some dyspeptic symptoms of a general nature).

Then continues this *teacher of homœopathy:* "We see from such comparisons that superficial prescribing will not do, and the totality of the symptoms means the pathological state which we have before us, be it a functional or already an abnormal organic one. We may be lost in a wilderness of symptoms, if we fail to consider the pathological characteristic which gives us the key-note to all the other symptoms. Thus, and only thus, our Materia Medica must be studied, as in no other manner [does] its study become easy, its strict application more definite."

"Superficial prescribing will not do," says this learned homœopath; and by superficial prescribing, we take it, he means "*symptom-covering.*" Now, it has just been shown that the totality of the symptoms includes the pathological condition of the patient; the pathological condition told in the simple, truthful language of the symptoms (felt and seen), untrammelled by any hypothesis. How then can "symptom-covering" be "superficial prescribing;" is not the term more applicable to him who prescribes on a pathological hypothesis?

Which is most superficial, to study every case as a new and separate disease, giving due importance to *all* symptoms; or to treat all cases of the same (nosological) disease as similar, basing such treatment upon a few general features common to all and ignoring the many features wherein they differ? What is the practice of these would be pathologist and teachers? They are those who see nothing in a gonorrhœa or a leucorrhœa or an otorrhæa but the discharge; this they endeavor to dry up, modelling their treatment after the foolish ostrich, who hides only his head, hoping that his body may not be seen. It is said that a professor in an homœopathic institution teaches his pupils that Phosphorus is *the* remedy for broncho-pneumonia, and Bryonia *the* remedy for pluro-pneumonia! There is indeed a kind of "superficial prescribing" that will not do,—*nor heal!!*

"Superficial prescribing will not do, and the totality of the symptoms means the pathological state," says our teacher. This, put in plain Anglo-Saxon means that quinia is pathologically the remedy for intermittent; morphia, for neuralgias and kindred pains; cathartics, for constipation. Carry out this list fully, and you have the precept and practice of the liberal-minded, would-be pathologist of today, who is often wrongly called by the honorable name of homœopathist.

"Liver and spleen enormously enlarged and deranged, is the keynote to the dyspepsia curable by *Nitric Acid*," says our teacher. Here the hypertrophy of liver and spleen is the pathological keynote, around which what would otherwise be a "wilderness of symptoms," to this mock-healer, are now evenly and orderly arranged. As Nitric Acid has never, so far as therapeutists are aware, caused any hypertrophy, "enormous" or otherwise, of spleen or liver, it will be seen that our scientific friend bases his "pathological key-note," without which he would "be lost in a wilderness of symptoms, upon a clinical or empirical hint. Truly, a noble path to guide one out of a "wilderness of symptoms;" it has only been trodden for some few thousand years.

"It is in the nature of the science of pathology that it always *ought* to be in advance of one certain knowledge regarding the treatment of disease," declares Dr. Aitkin, a high authority—for some. If this be acknowledged, then the question of the use of pathology in homæopathic therapeutics is settled; *for pathology is not "in advance of our certain knowledge regarding the treatment of disease;"* nor it will ever be in that position, *if our law be true.* And those who believe pathology is thus in advance, cannot believe in the truth of our law.

The gentlemen, whose pathological vagaries we have been noticing, once declared that: "when men like Dr. Carroll Dunham, and Dr. ———, make an assertion—men who never left anything undone in their lives, I believe it. Dr. Dunham knew his pathology as well as any one." Having such an high and just opinion of Dr. Dunham, (who please remember, "knew his pathology") we hope this gentleman will listen now to him, "who being dead yet speaketh."

Dr. Dunham wrote* "And those of our school who insist upon pathology as a *basis* of therapeutics, who look upon the single objective symptom and its nearest organic origin as the subject for treatment, and who deride the notion of prescribing upon the totality of the symptoms, and claim to be more than mere symptom coverers, in that they discover and aim to remove the *cause* of the disease—these colleagues are as false in their pathology, according to

* See, Homœopathy, the Science of Therapeutics, p. 114.

the highest old-school authorities, as they are faithless to the doctrines, and impotent as to the successes of the founder of the homœopathic school."

